# Mitochondrial Lon Peptidase 1 Controls Diaphragm and Lung Development in a Context-Dependent Manner

**DOI:** 10.70322/jrbtm.2025.10008

**Published:** 2025-08-20

**Authors:** Le Xu, Chunting Tan, Nicole Talaba, Andrew Sou, Yufeng Shen, Wendy K. Chung, David J. McCulley, Xin Sun

**Affiliations:** 1 Department of Pediatrics, School of Medicine, University of California San Diego, La Jolla, CA 92093, USA; 2 Department of Systems Biology, Columbia University Irving Medical Center, New York, NY 10032, USA; 3 Department of Biomedical Informatics, Columbia University Irving Medical Center, New York, NY 10032, USA; 4 JP Sulzberger Columbia Genome Center, Columbia University Irving Medical Center, New York, NY 10032, USA; 5 Department of Pediatrics, Boston Children’s Hospital and Harvard Medical School, Boston, MA 02115, USA; 6 Department of Cell and Developmental Biology, University of California San Diego, La Jolla, CA 92093, USA

**Keywords:** CDH, Diaphragm, Lung, Mitochondria, LONP1, SHH, FGF10

## Abstract

Congenital Diaphragmatic Hernia (CDH) is a rare neonatal disorder causing diaphragmatic defects and cardiopulmonary hypoplasia, traditionally attributed to mechanical compression from organ herniation. However, emerging evidence suggests genetic mutations may independently impair lung development, prompting debate over CDH etiology. Here, we investigated the requirement of mitochondrial function guarded by LON peptidase 1 (*Lonp1*), a CDH risk gene, in either diaphragm or lung development. *Lonp1* loss in skeletal muscles of the diaphragm led to its thinning and membranization, recapitulating the pathology of sac-type CDH. On the other hand, lung-specific inactivation caused severe hypoplasia with defective branching morphogenesis, independent of diaphragm anomalies. Molecularly, *Lonp1* disruption dysregulated key transcription factors and signaling pathways known to be critical for early lung development. Our findings here revealed that mitochondrial defects contribute to the pathogenesis of CDH in an organ and cell type specific manner, opening new avenues for drug and therapeutic development.

## 1. Introduction

Congenital Diaphragmatic Hernia (CDH) is a rare but severe developmental disorder affecting approximately 1 in 2500 live births, characterized by incomplete diaphragm development during embryogenesis. As a result, abdominal organs, such as the liver and intestine, herniate into the thoracic cavity either naked or enclosed by a non-muscularized diaphragmatic sac (sac-type CDH) [1], which profoundly impacts the growth of the heart and lung. Pulmonary hypoplasia remains the leading cause of morbidity and mortality in CDH patients, with survival rates ranging from 50–70% despite advances in neonatal care. Due to the coincidence of lung growth and the progression of diaphragmatic herniation, lung underdevelopment in CDH has long been attributed to mechanical compression by herniation, making the diaphragm defect the primary driver of lung abnormalities. Successful therapeutic treatments have been established to promote lung expansion and growth in fetuses with severe CDH through elevating the intrapulmonary mechanical force [2].

Genetic manipulation in mouse models revealed that cross-organ mutations in genes involved in fundamental cellular processes, such as gene transcription, signaling transduction and cell cycle regulation, can recapitulate key pathological features of CDH. Studies report simultaneous diaphragmatic and pulmonary abnormalities, including inactivation of multiple transcription factors GATA4, FOG2, COUP-TFII and WT1 [3–6], and disruption of retinoic acid signaling [7–9]. Intriguingly, in the recent studies of chromatin regulator SIN3A and transcription factor MYRF, though both diaphragmatic and pulmonary defects occur in either mutant mice, lung anomalies can be further observed in the absence of hernia induced mechanical compression [10,11]. Those findings suggest that lung hypoplasia in CDH may not solely be a physical consequence of herniation but could involve intrinsic genetic defects within the lung itself, in concert with or independent of diaphragmatic anomalies. This additional genetic component may explain why hernia repair does not always lead to recovery of lung function.

Known as the powerhouse and key signaling regulator in the cell, mitochondria play a variety of critical roles in controlling organogenesis. Recent studies in lung epithelium revealed that healthy mitochondria are required for maintaining alveolar cell stemness [12–15]. Mitochondrial dysfunction significantly impairs the differentiation of myoblast cells [16], which are the building blocks for skeletal muscle formation and growth in the diaphragm. Despite those isolated findings, the extent to which degree and how mitochondria related defects affect lung and diaphragm development in the context of CDH remains largely unexplored. Recent human genetic studies revealed that a gene named *LONP1*, which encodes a protease localized explicitly in the mitochondrial matrix and dictates mitochondrial protein quality control, was ranked as one of the top frequently mutated genes in CDH patients [17]. Inactivation of *Lonp1* in early mouse lung development led to defective branching morphogenesis and airway cell differentiation, suggesting a lung intrinsic role of mitochondria in the pathogenesis of CDH [18].

In this study, through conditional knock out of *Lonp1* as a genetic handle, we addressed the role of mitochondria in different lung and diaphragm cell types for the mouse modeling of CDH. We found that *Lonp1* inactivation in skeletal muscle precursors of the diaphragm via *Pax3*^*Cre*^ results in severe diaphragmatic thinning and muscularization defects reminiscent of sac-type CDH. Epithelial inactivation of *Lonp1* via *Shh*^*Cre*^ leads to profound lung hypoplasia characterized by defective branching morphogenesis and disrupted proximal-distal patterning, independent of diaphragmatic defects. On the contrary, no developmental phenotypes of either diaphragm or lung were observed in the mutant mice where *Lonp1* was inactivated in the mesenchymal cell compartments of both organs. Transcriptomic analysis reveals that *Lonp1* modulates key transcription factors and signaling pathways, including the SHH-FGF10 signaling axis in epithelial-mesenchymal crosstalk. Our findings collectively uncovered the organ- and cell type-specific roles of mitochondria in the etiology of CDH, and provided insights for advancing therapeutic strategies using mitochondria as potential targets.

## 2. Materials and Methods

### 2.1. Mice

*Shh*^*cre*^, *Pax3*^*cre*^, *Tbx4rtTA;TetOcre* and *Lonp1*^*flox*^ mice have been described previously [17,19–21]. Embryos used in this study were harvested from time-mated mice, counting noon of the day when the vaginal plug was found as E0.5. All mice were in a B6 background. Littermates were used as controls in all experiments. Both males and females were randomly assigned to experimental groups. All mice were housed in facilities accredited by the American Association for Accreditation of Laboratory Animal Care (AAALAC) at the University of California, San Diego. The Institutional Animal Care and Use Committee (IACUC) approved all animal husbandry and experiments.

### 2.2. Tissue Preparation and Immunofluorescent Staining

Trachea and lungs from embryos were fixed in 4% paraformaldehyde (Electron Microscopy Sciences, Hatfield, PA, USA) diluted in PBS overnight at 4 °C. Samples were embedded in either paraffin or OCT (Electron Microscopy Sciences) for sectioning. Antigen retrieval was performed before serum-mediated blocking using high-PH retrieval buffer (10 mM Tris, 1 mM EDTA, pH 9.0). Primary antibodies with final concentrations used for immunofluorescence staining are: rabbit anti-SOX2 polyclonal antibody [8 mg/mL] (NB110–37235, Novus Biologicals, Centennial, CO, USA), mouse anti-SOX9 monoclonal antibody [5 mg/mL] (AMAB90795, Sigma, Tokyo, Japan), mouse anti-aSMA monoclonal antibody [5 mg/mL] (A2547, Sigma), mouse anti-E-cadherin monoclonal antibody [8 mg/mL] (610181, BD Transduction Laboratories, NJ, USA), rabbit anti-E-cadherin polyclonal antibody [5 mg/mL] (3195, Cell Signaling Technology, Danvers, MA, USA), rabbit anti-FOXA1 monoclonal antibody [5 mg/mL] (ab173287, Abcam, Cambridge, UK) and rabbit anti-FOXA2 monoclonal antibody [5 mg/mL] (ab108422, Abcam). The following secondary antibodies were used with final concentration: Cy3-conjugated goat anti-mouse IgG [2 mg/mL] (115–165-003, Jackson ImmunoResearch, West Grove, PA, USA), Cy3-conjugated goat anti-rabbit IgG [2 mg/mL] (111–165-144, Jackson ImmunoResearch), AF488-conjugated goat anti-mouse IgG [2 mg/mL] (A-11001, Invitrogen, Carlsbad, CA, USA), AF488-conjugated goat anti-rabbit IgG [2 mg/mL] (A-11008, Invitrogen). All images were acquired on the ZEISS AxioImager 2, except for slide scans on Olympus SLIDEVIEW VS200. 20X or 40X IF images were used to quantify cells labeled by specific markers. At least 3 sections per mouse and 3 mice per genotype were analyzed for each condition.

### 2.3. Lung Explant Culture

Lungs from control and *Shhcre;Lonp1* mutant embryos were harvested at E11.5. Lungs were placed on a Nucleopore Trak-Etch membrane (Whatman; 8 μm) and cultured at the air/liquid interface with DMEM-F12 and Penicillin-Streptomycin (15140122, Gibco, MA, USA). To antagonize FGF10 signaling, SU5402 (SML0443, Sigma) was added at a final concentration of 10 μM. DMSO was used as a diluent control. Lungs were cultured at 37 °C in 5% CO_2_ for 48 h, and samples were harvested and analyzed at 0, 24 and 48 h.

### 2.4. Quantitative PCR (qPCR)

Total RNA from embryonic and adult lungs were extracted using Trizol (Invitrogen) and RNeasy Micro RNA extraction kit (74104, Qiagen, Germany). Reverse transcription was then carried out to obtain corresponding cDNA using iScript Select cDNA Synthesis Kit (Bio-Rad, Hercules, CA, USA). qPCR master mix was prepared using SYBR Green reagents (Bio-Rad), and amplification signals were obtained by CFX ConnectTM system (Bio-Rad). At least three biological replicates were assayed for each gene unless otherwise noted. Primers used for qPCR analysis are listed in Supplemental Table S1.

### 2.5. Bulk RNA-Seq and Data Analysis

Total RNA from embryonic lungs at E13.5 were extracted as described above. cDNA libraries were constructed using Illumina TruSeq RNA Library Prep Kit V2 (Illumina, San Diego, CA, USA) and sequenced on the HiSeq4000 platform (Illumina) at the Institute for Genomic Medicine (IGM) at UCSD. FASTQ files were aligned to the mouse reference genome (mm10) using Bowtie2 [22] with default settings. Differential gene expression analysis was performed using Cufflinks [23]. Heatmaps and volcano plots were generated by ggplot2 (version 3.3.2).

### 2.6. Data Availability

Raw and processed files from bulk RNA-seq have been uploaded to the GEO database under accession number GSE230334.

### 2.7. Quantification and Statistical Analysis

For quantification using sections, 3 sections per sample were analyzed. The sections were selected from equivalent depths from sample to sample. Student’s *t* test was used for calculating significance. All statistical analyses were performed using Prism 9 (GraphPad). Significance in the Figures: ns, not significant, *p* > 0.05; ** for *p* < 0.01; Error bars represent mean ± SD.

## 3. Results

### 3.1. Lonp1 Is Required for Skeletal Muscle Formation in the Diaphragm

**Figure 1.**
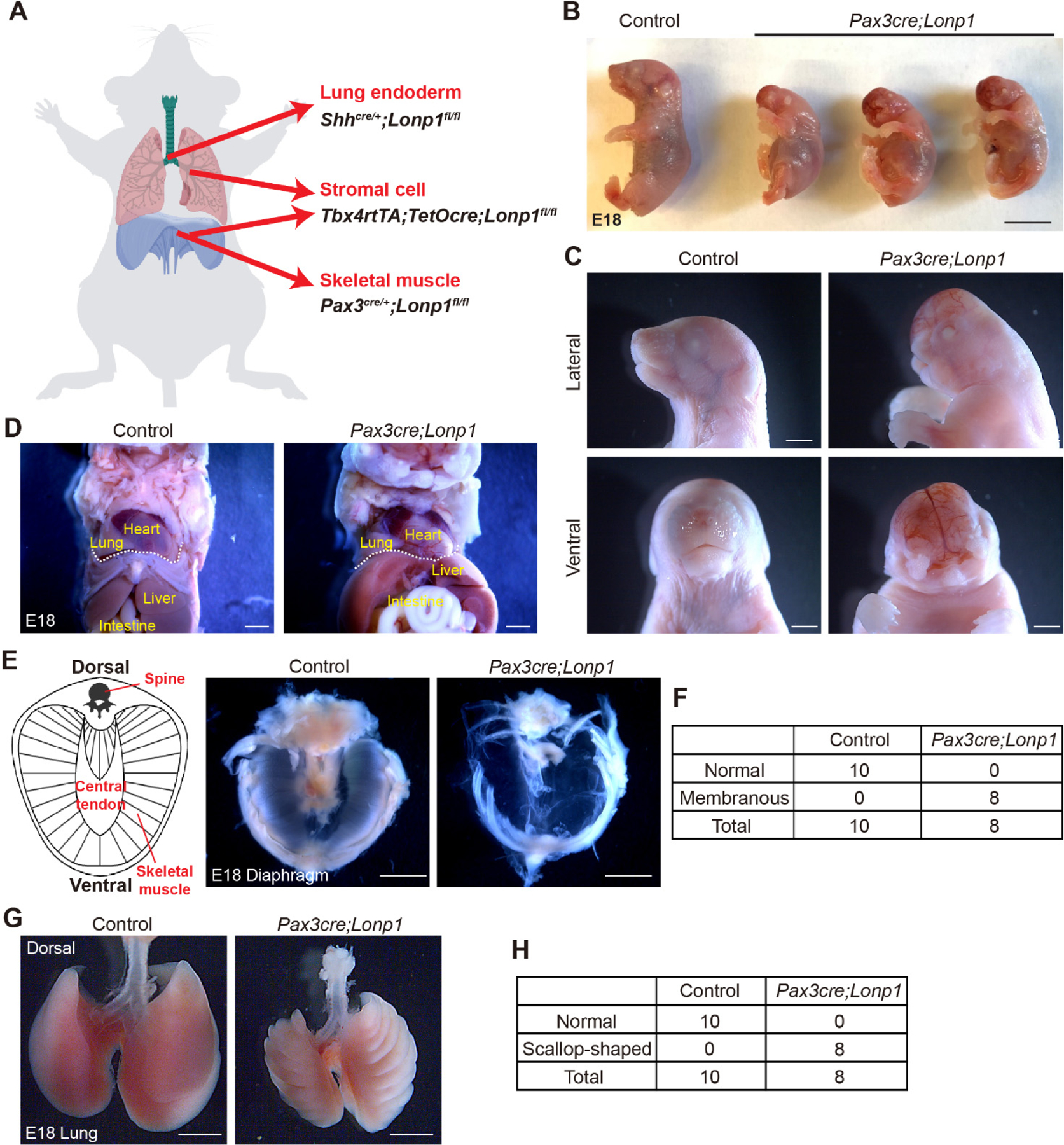
*Lonp1* is required for skeletal muscle development in the diaphragm. (**A**) Diagram showing the tissue-specific inactivation of *Lonp1*. (**B**,**C**) Embryonic (**B**) and craniofacial (**C**) phenotypes of *Pax3cre;Lonp1* and control mice at E18. Scale bars, 0.5 cm in (**B**) and 1 mm in (**C**). (**D**) Anatomic localizations of internal organs of *Pax3cre;Lonp1* and control mice at E18. The diaphragm is marked by the dashed white line. Scale bars, 1 mm. (**E**,**F**) Diaphragms of *Pax3cre;Lonp1* and control mice at E18. The cartoon on the left shows major tissues in the normal diaphragm. Scale bars, 0.2 cm. Quantifications were shown in (**F**). (**G**,**H**) Lungs of *Pax3cre;Lonp1* and control mice at E18. Scale bars, 0.2 cm. Quantifications were shown in (**H**).

The diaphragm is a contracting dome-shaped muscle tissue that controls breathing. To determine if *Lonp1* plays essential roles in the development of the diaphragm, we conditionally inactivate *Lonp1* in skeletal muscle cells by generating *Pax3*^*cre/+*^*;Lonp1*^*fl/fl*^ (hereafter referred to as *Pax3cre;Lonp1*) mutant mice (Figure 1A). All newborn pups of *Pax3cre;Lonp1* died shortly after birth. Compared to their littermate controls (*Pax3*^*cre/+*^*;Lonp1*^*fl/+*^), mutant embryos at the perinatal stage, Embryonic day (E) 18, exhibited overall smaller bodies in size (Figure 1B). We found striking craniofacial malformations in the *Pax3cre;Lonp1* mutant mice, including the lack of development of the entire nose and mouth organs (Figure 1C).

Anatomical analysis in the chest and abdominal organs suggested no evidence of diaphragmatic herniation in *Pax3cre;Lonp1* mutants (Figure 1D). However, we found drastic tissue malformation in the diaphragms of *Pax3cre;Lonp1* mutants. In contrast to the normal diaphragm composed of skeletal muscle cells in the periphery surrounding the central tendon, the *Lonp1*-deficient diaphragm developed into a thin and nearly transparent membrane with complete loss of skeletal muscle cells (Figure 1E,F). This phenotype recapitulates key features of sac-type CDH patients, in which the defect is not a hole but a thinning and/or incomplete muscularization of the diaphragm [24]. Though *Pax3* is barely expressed throughout lung development (Figure S1) [25], we found lungs in the *Pax3cre;Lonp1* mutants developed with scallop-shaped edges (Figure 1G,H). It is possible that, in the *Pax3cre;Lonp1* mutants, lungs developed in a “static” position restricted by the chest wall and ribs that don’t develop or move sufficiently. Collectively, these data suggest that *Lonp1* is required for skeletal muscle differentiation in both craniofacial and diaphragmatic tissues.

### 3.2. Lonp1 Is Required for Lung Branching Morphogenesis

**Figure 2.**
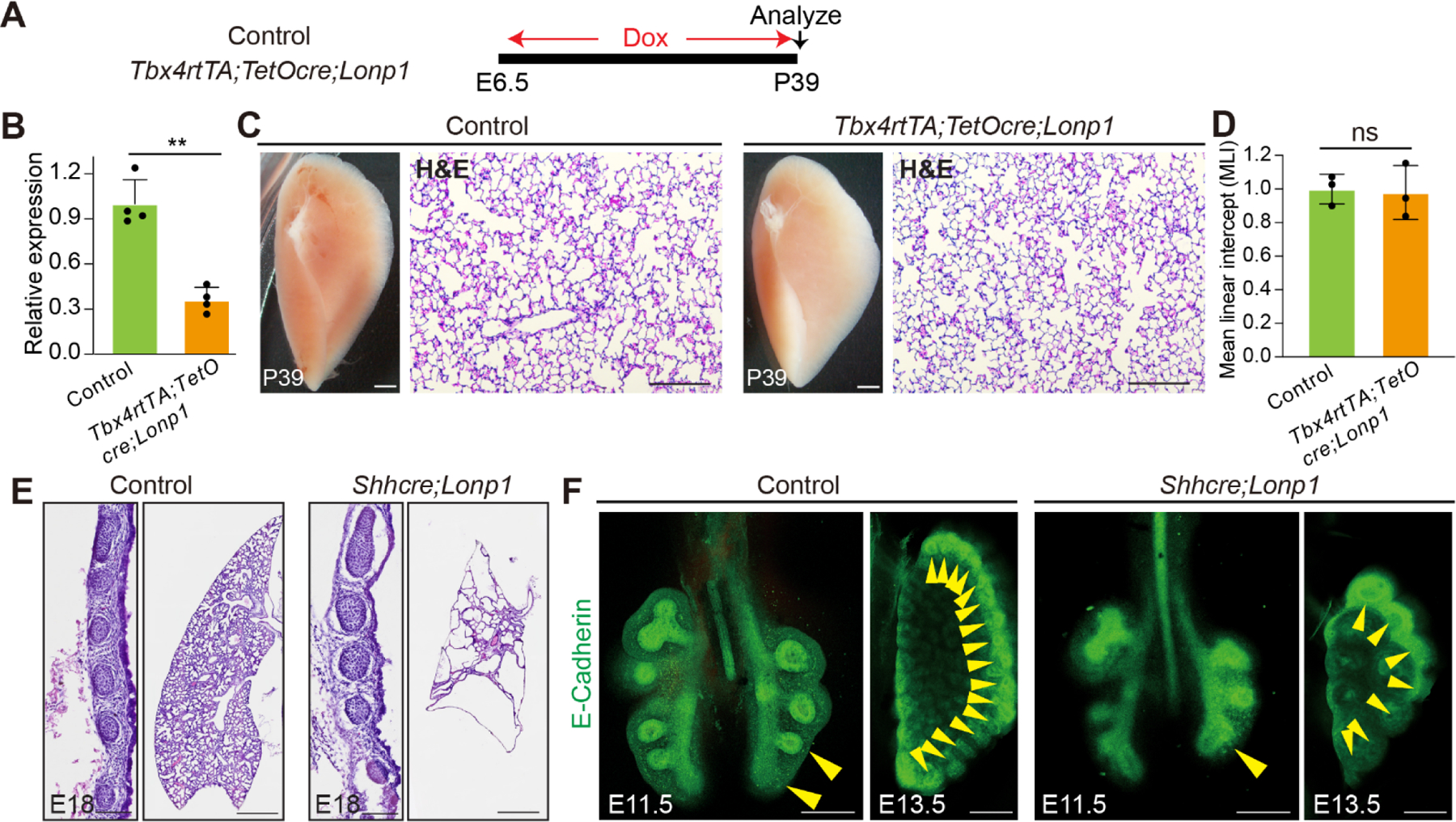
*Lonp1* is required for lung development. (**A**) Diagram showing the inactivation of *Lonp1* in lung mesenchyme. (**B**) RNA levels of *Lonp1* in the lungs of *Tbx4rtTA;TetOcre;Lonp1* and control mice *were* measured by RT-qPCR. (**C**) Lung morphologies of *Tbx4rtTA;TetOcre;Lonp1* and control mice were measured by brightfield microscope and H&E staining. Scale bars, 1 mm in brightfield, 100 μm in H&E. (**D**) MLI quantifications of lungs of *Tbx4rtTA;TetOcre;Lonp1* and control mice. (**E**) Trachea and lung morphologies of *Shhcre;Lonp1* and control mice at E18. Scale bars, 100 μm for trachea, 200 μm for lung. (**F**) Whole-mount E-cadherin staining of airway epithelium in *Shhcre;Lonp1* and control mice at E11.5 and E13.5. Branching tips were labeled by yellow arrowheads. Scale bars, 50 μm.

Previous studies showed that *Shh*^*cre*^-mediated inactivation of *Lonp1* in lung endoderm led to drastic lung hypoplasia with the replacement of lobes by cyst-like structures [17]. To comprehensively evaluate the intrinsic roles of *Lonp1* during lung development, we inactivated *Lonp1* in additional cell lineages by generating *Tbx4rtTA;TetOcre;Lonp1*^*flox/flox*^ (hereafter *Tbx4rtTA;TetOcre;Lonp1*) mice. Continuous doxycycline administration starting at E6.5 (Figure 2A), known to trigger cre activity broadly in the lung mesenchyme and endothelium, led to efficient reduction of *Lonp1* transcripts (~70% reduction, Figure 2B). Interestingly, we found that mutant mice at the adult stage Postnatal day 39 (P39) are viable with no obvious abnormalities in lung structure by histology analysis (Figure 2C,D). *Tbx4rtTA;TetOcre* had been shown to be active in the mesothelial cells of diaphragm [11]. Similar to observations in lung, no diaphragmatic defects were noticed in the *Tbx4rtTA;TetOcre;Lonp1* mutant mice (Figure S2). These data suggest that *Lonp1* is dispensable for the development of mesenchymal/mesothelial cells in the lung and diaphragm, respectively.

Next, we focused our studies on the roles of Lonp1 in lung endoderm. At the perinatal stage E18, *Shh*^*cre/+*^*;Lonp1*^*flox/flox*^ (hereafter *Shhcre;Lonp1*) mutant mice exhibited multiple malformations in the trachea and lung. In comparison to control trachea, we found a strong reduction of cellularity in the stromal tissues that surrounds the cartilage in the *Shhcre;Lonp1* mutants (Figure 2E, left panels). HE staining showed that there is largely an absence of alveolar tissues in the *Shhcre;Lonp1* mutant lung (Figure 2E, right panels). Wholemount immunostaining with E-cadherin to highlight the epithelium showed that lung branching defects manifested as early as E11.5, at the start of secondary branching morphogenesis (Figure 2F, left panels). At E13.5, mutant lungs exhibited reduced tip number and dilated tips (Figure 2F, right panels). Taken together, these in vivo data suggest that *Lonp1* selectively functions in the endoderm to control lung branching morphogenesis.

### 3.3. Lonp1 Is Required for Lung Proximal-Distal Patterning

**Figure 3.**
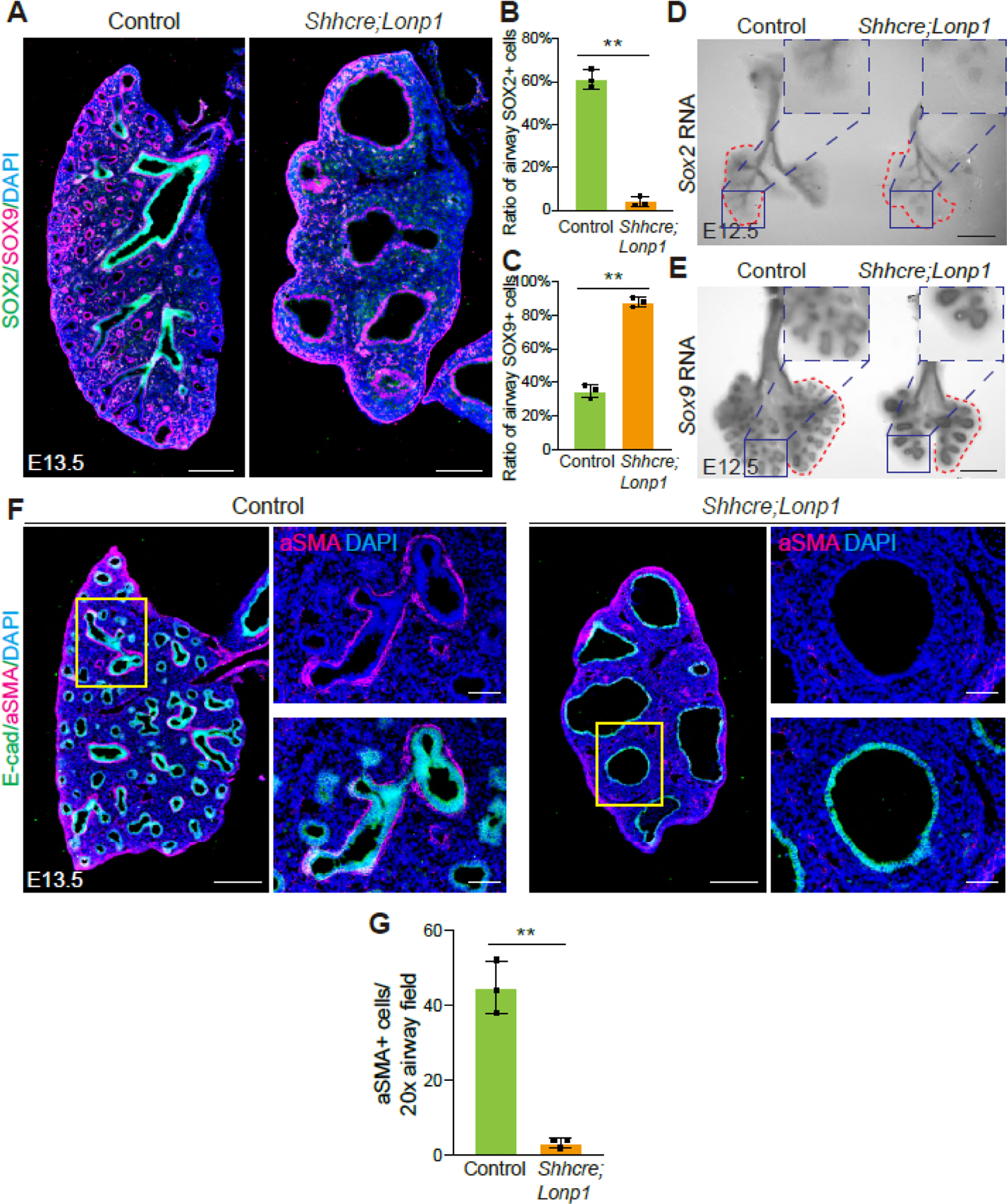
*Lonp1* is required for lung proximal-distal patterning. (**A**–**C**) Representative images of SOX2 and SOX9 staining in the lungs of *Shhcre;Lonp1* and control mice at E13.5. Scale bars, 200 μm. Quantifications of SOX2 (**B**) and SOX9 (**C**) were shown, respectively. (**D**,**E**) *In situ* hybridization analysis of *Sox2* (**D**) and *Sox9* (**E**) RNAs in the lungs of *Shhcre;Lonp1* and control mice at E12.5. Scale bars, 200 μm. Selected airway regions were boxed and magnified on the top right. (**F**,**G**) Representative images of aSMA staining in the lungs of *Shhcre;Lonp1,* and control mice at E13.5 (**F**). Selected airway regions were boxed and magnified on the right: scale bars, 200 μm. Quantifications were shown in (**G**).

To determine if *Lonp1* plays a role in regulating proximal-distal patterning of the lung, we examined the expression patterns of *Sox2* (marker for proximal airway epithelium) and *Sox9* (marker for distal airway epithelium) at both the protein and RNA levels. In contrast to the mutually exclusive staining of both SOX2 and SOX9 in the control lungs at E13.5, SOX9 is broadly enriched in the enlarged epithelium of *Shhcre;Lonp1* mutants without the presence of SOX2 (Figure 3A–C). Detection of RNA transcripts by *in situ* hybridization demonstrated that, in the E12.5 mutant lung, *Sox2* RNA was primarily restricted to the extrapulmonary bronchi, while in the control lungs these RNA signals went deep into the intrapulmonary airways (Figure 3D). *Sox9* RNA was normally restricted in the distal epithelium, but was expanded to most of the lung epithelium in the *Shhcre;Lonp1* mutants (Figure 3E). Given extensive cell-cell interactions between airway epithelium and its surrounding mesenchymal cells, we examined the differentiation of airway smooth muscle cells. In accordance with the absence of proximal SOX2+ epithelial cells, aSMA+ smooth muscle cells were significantly decreased in the *Shhcre;Lonp1* mutant lungs (Figure 3F,G). These results together suggest that *Lonp1* is essential for lung proximal-distal epithelium patterning.

### 3.4. Dysregulation of Primordial Transcription Factors and Shh-Fgf10 Signaling Axis in Lonp1 Mutant Lungs

**Figure 4.**
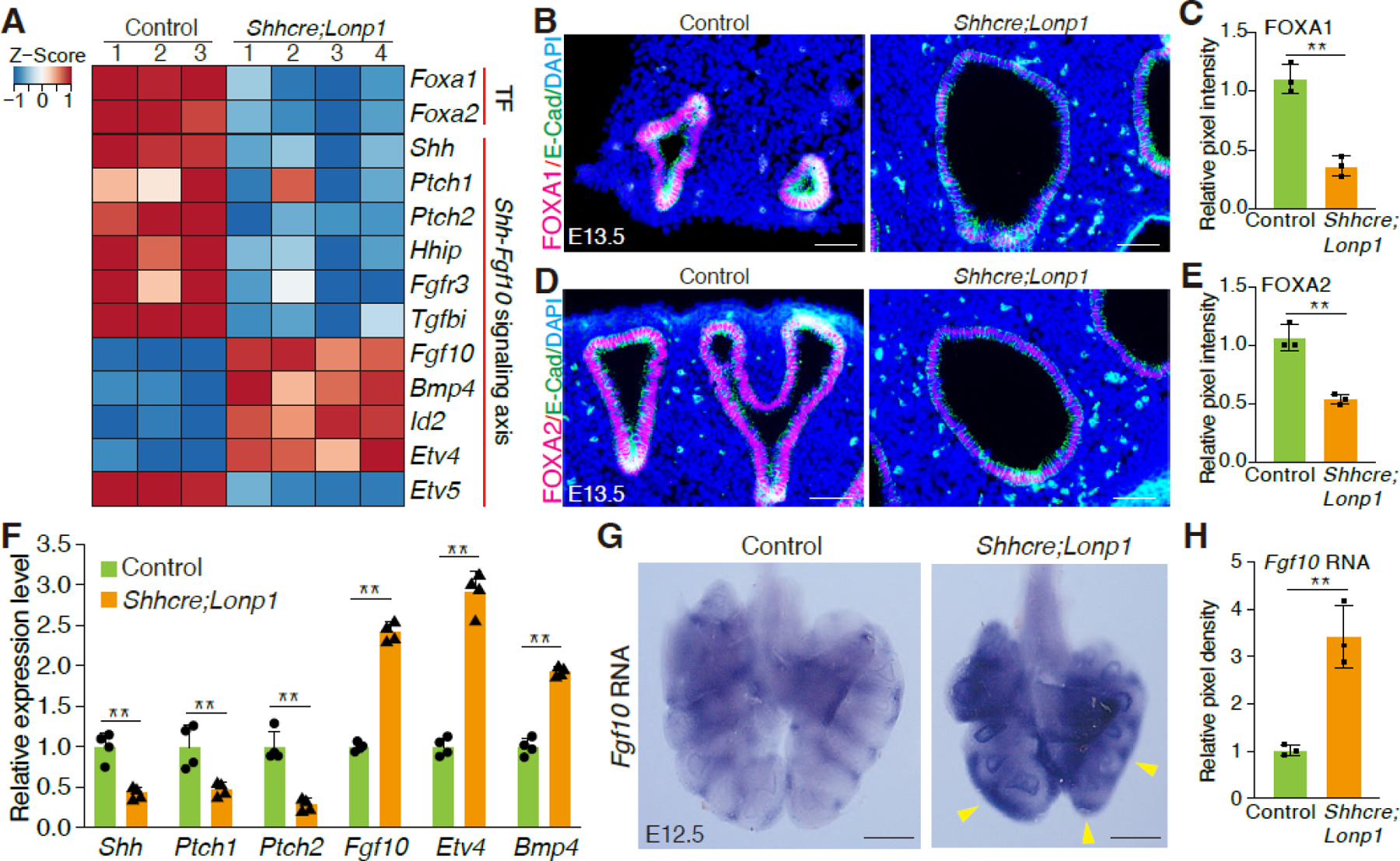
Loss of *Lonp1* impairs lung epithelium-to-mesenchyme crosstalk. (**A**) Expression profiling of selected genes in the lungs of *Shhcre;Lonp1* and control mice by bulk RNA-seq. (**B**,**C**) Representative images of FOXA1 staining in the lungs of *Shhcre;Lonp1* and control mice at E13.5 (**B**). Protein expression levels measured by pixel intensity were quantified in (**C**). Scale bars, 200 μm. (**D**,**E**) Representative images of FOXA2 staining in the lungs of *Shhcre;Lonp1* and control mice at E13.5 (**D**). Protein levels measured by pixel intensity were quantified in (**E**). Scale bars, 200 μm. (**F**) RNA levels of selected genes in the lungs of *Shhcre;Lonp1,* and control mice were measured by RT-qPCR. (**G**,**H**) *In situ* hybridization analysis of *Fgf10*. Elevated RNA signals in the distal lung were labeled by yellow arrowheads. (**G**) RNAs in the lungs of *Shhcre;Lonp1* and control mice at E12.5. RNA levels measured by pixel intensity were quantified in (**H**). Scale bars, 200 μm.

To investigate the molecular mechanisms that led to lung hypoplasia following epithelial inactivation of *Lonp1*, we performed transcriptomic analysis of *Lonp1* mutant and control lungs at E13.5. Expression profiling revealed that two transcription factors, *Foxa1* and *Foxa2*, were down-regulated in the *Lonp1* mutant lungs (Figure 4A). Immunostaining confirmed their reduction in the epithelial cells at the protein level (Figure 4B–E). Notably, FOXA1/FOXA2 were considered the primordial transcription factors controlling early lung development. Co-inactivation of *Foxa1; Foxa2* led to reduced branching and decreased expression of key epithelium signaling gene *Shh* [26–28]. Consistent with these findings, in the *Shhcre;Lonp1* mutant, the expression of *Shh* and its mesenchymal targets *Ptch1, Ptch2* and *Hhip* was decreased compared to *Shhcre;Lonp1* heterozygous control (Figure 4A). Conversely, the expression of SHH repressed mesenchymal signaling gene *Fgf10*, and several of the FGF10 downstream targets, including *Etv4*, *Bmp4, and Id2,* were up-regulated (Figure 4A). Curiously, *Etv5*, another FGF10 downstream target, was downregulated, suggesting complex regulation of signaling. These signaling-related changes were further validated by qRT-PCR (Figure 4F). By *in situ* hybridization, there was a clear expansion of *Fgf10* in the mesenchyme surrounding the tips in the *Lonp1* mutants (Figure 4G,H). Since *Foxa1*/*Foxa2* and SHH-FGF feedback loop genes are critical for branching morphogenesis [26–28], changes in these genes may contribute to the branching defects observed in *Shhcre;Lonp1* mutant mice.

To determine if the elevation in FGF10 signaling in the *Lonp1* mutants contributes to the branching phenotypes, we attenuated FGF10 activity using a pharmacological FGFR-selective inhibitor, SU5402, in the explant lung culture system. Treatment of control lungs with SU5402 medium was effective in robustly decreasing the tip number to 3 after 48 h of culture (Figure S3A,B). In contrast, repression of branching by SU5402 at the same concentration was significantly attenuated in the Lonp1 mutant lungs, leading to around 7 tips after 48 h of culture (Figure S3A,B). These results demonstrate that the elevated FGF10 signaling in the *Lonp1* mutants contributes to the lung branching inhibition, and that *Lonp1* promotes lung branching partially through maintaining the SHH-FGF feedback loop in the epithelial-mesenchymal crosstalk.

## 4. Discussion

Our lack of knowledge on the cellular origins of this catastrophic disease hinders the advancement of precise therapeutics for CDH patients. Our findings here revealed that mitochondrial dysfunction is a genetic cause of CDH, impairing either diaphragm or lung development in an organ- and cell type-specific manner. Loss of mitochondrial protease LONP1 in skeletal muscle cells of the diaphragm and epithelial cells of the lung led to CDH-featured phenotypes, respectively, while its inactivation in mesenchymal cells of both organs confers no visible developmental defects. It’s all remarkable given the housekeeping functions of mitochondria in the cell and the substantial role of mesenchymal cells as the structural support for those mobile organs. How those context-dependent roles of mitochondria are established and maintained across organs is of great interest for future study.

Sac-type CDH, recapitulated in the *Pax3cre;Lonp1* mutant mice, is a distinct subtype of CDH characterized by the presence of a membranous sac covering the diaphragmatic defect, as opposed to the more common canonical CDH. Sac-type CDH is associated with a better prognosis than canonical CDH, with survival rates exceeding 90% in some cohorts, likely due to reduced herniation and less severe pulmonary hypoplasia [29]. Consistently, in *Pax3cre;Lonp1* mutant mice, no abdominal organs herniate through the defective diaphragm, and the lung size and tissues remain relatively normal except for the morphological changes in the scallop shape. As *Pax3* is not expressed in the lung and the rhythmic grooves along the dorsal-lateral surface of the mutant lung align with the rib architecture, this phenotype could reflect a compensatory overgrowth or folding of lung tissue constrained by an immobile chest wall, as the lung attempts to fill the available space in between the ribs without the stretching normally provided by muscularized diaphragm.

Proper patterning along the proximal-distal axis is essential for coordinated branching morphogenesis and alveolar differentiation. Impaired patterning may reduce distal epithelial progenitor cells and defective airway formation, both of which compromise pulmonary structure and function. In the context of CDH, where pulmonary hypoplasia is a hallmark feature, aberrant proximal-distal specification may exacerbate the developmental arrest of the lung. Our findings suggest that loss of *Lonp1* disrupts this key developmental process, potentially contributing to the hypoplastic phenotype observed in CDH by limiting the expansion and differentiation of the distal lung epithelium necessary for alveolar development and gas exchange capacity.

The lung phenotype of *Shhcre;Lonp1* mutants shares striking similarities with that of *Foxa1/Foxa2* double mutants [28]. Characterized as the two pioneer transcription factors required for early lung development, FOXA1 and FOXA2 are able to bind enhancers of key morphogenetic genes, including the signaling molecule *Shh* [30]. Consistently, the downregulation of *Foxa1* and *Foxa2* in *Lonp1*-deficient lungs correlates with reduced *Shh* expression and elevated *Fgf10* signaling, recapitulating key molecular features of *Foxa1/Foxa2* compound mutant lungs. The data suggest that *Lonp1* controls lung development, in part, by acting even upstream of FOXA1/FOXA2-mediated transcriptional networks. The molecular link between mitochondrial perturbations and nuclear epigenomic responses in the airway epithelial cells is of great interest and needs further exploration.

## 5 Conclusions

In summary, our work demonstrated that mitochondrial peptidase LONP1 controls diaphragm and lung morphogenesis in an organ- and cell type-specific manner. This dual-organ perspective significantly advances our understanding of the complexity of CDH etiology, offering new avenues for developing mitochondrial-targeted therapies for this devastating neonatal disorder.

## Supplementary Material

Supplementary

The following supporting information can be found at: https://www.sciepublish.com/article/pii/644. Figure S1. *Pax3* is rarely expressed in the mouse lung. Figure S2. Diaphragm development is not affected in *Tbx4rtTA;TetOcre;Lonp1* mutant mice. Figure S3. Loss of *Lonp1* impairs FGF10 signaling during lung development. Table S1. DNA sequences of primers used for RT-qPCR.

## Data Availability

Raw and processed files from bulk RNA-seq have been uploaded to the GEO database under accession number GSE230334.
