# Supplementary material for: Mitochondrial Lon Peptidase 1 Controls Diaphragm and Lung Development in a Context-Dependent Manner
Source: J Respir Biol Transl Med. Author manuscript; Available in PMC 2025 Oct 3. (PMC12490049; doi:10.70322/jrbtm.2025.10008)
Supplement: Supplementary [file NIHMS2106242-supplement-Supplementary.pdf]

Figure S1 Xu et al.

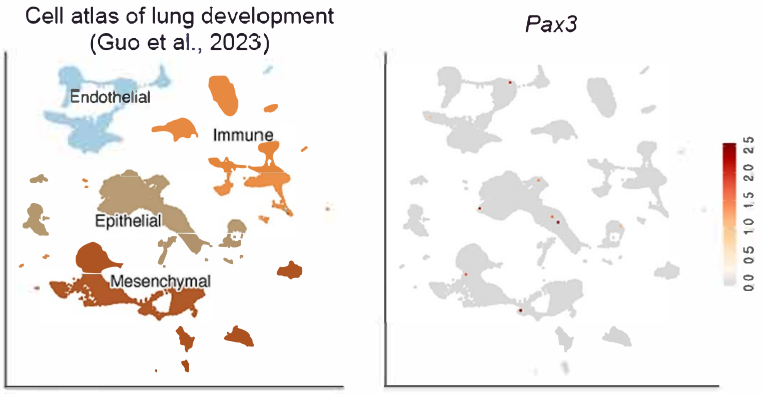

Figure S2 Xu et al.

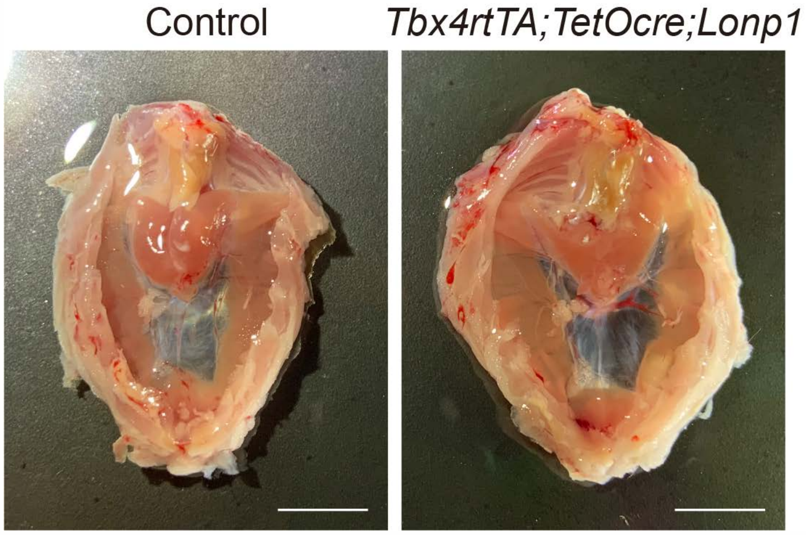

Figure S3 Xu et al.

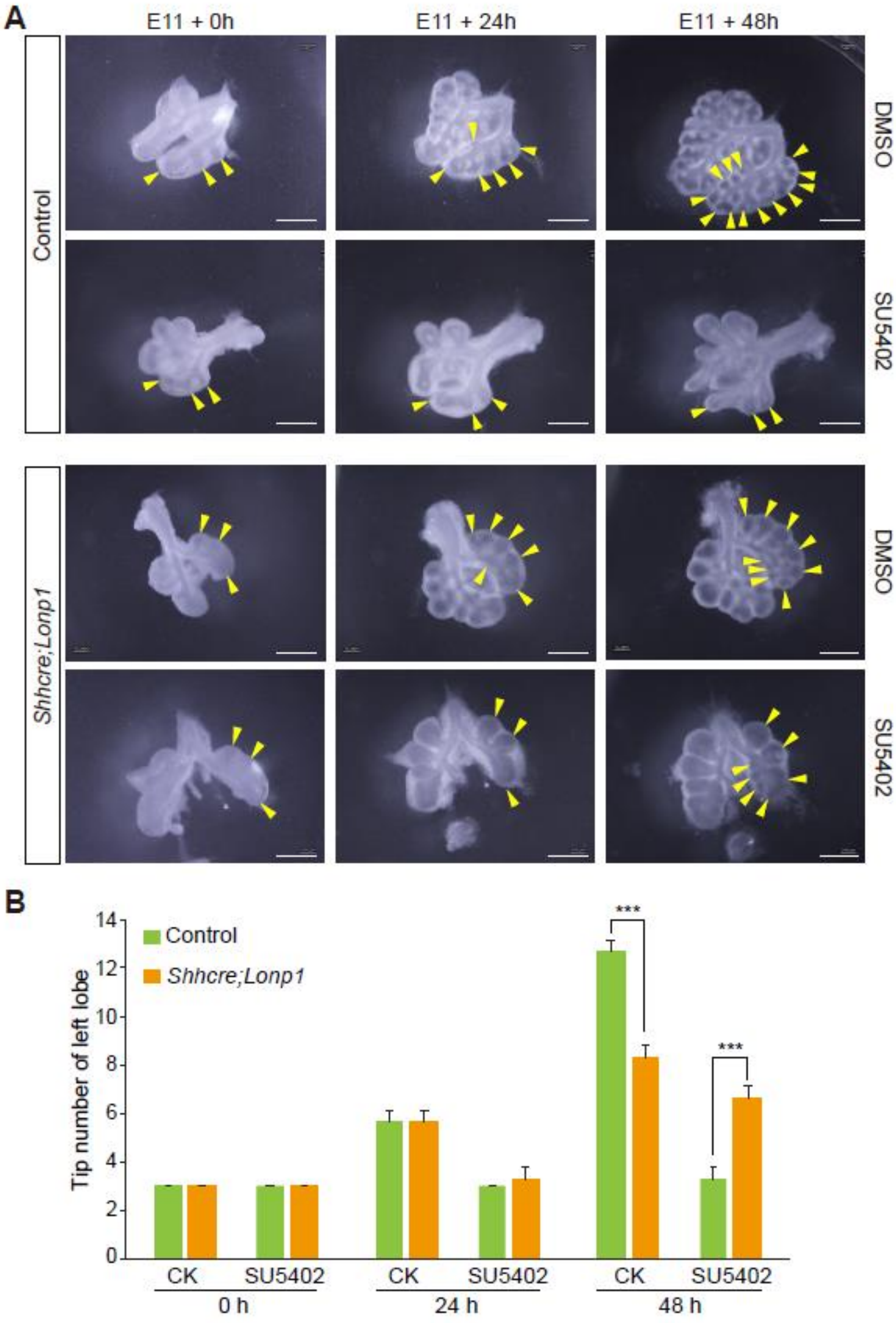

Supplementary Table S1

Gene	5' Primer	3' Primer
Shh	GGATGAGGAAAACACGGGAGCA	TCATCCCAGCCCTCGGTCACT
Ptch1	CCTCGCTTACAAACTCCTGGTG	TGATGCCATCTGCGTCTACCAG
Ptch2	GGCACTCACATCCGTCAACAAC	GAAGACGAGCATTACCGCTGCA
Fgf10	ATCACCTCCAAGGAGATGTCCG	CGGCAACAACTCCGATTTCCAC
Etv4	CACAGACTTCGCCTACGACTCA	GCAGACATCATCTGGGAATGGTC
Bmp4	GCCGAGCCAACACTGTGAGGA	GATGCTGCTGAGGTTGAAGAGG

Lonp1	CCAAGCATGTGATGGACGTGGT	GTCCAAGTTCTCATCACTCTGCC
Gapdh	CATCACTGCCACCCAGAAGACTG	ATGCCAGTGAGCTTCCCGTTCAG
